# Supplementary material for: Validation of a Genotype-Independent Hepatitis C Virus Near-Whole Genome Sequencing Assay
Source: Viruses. 2021 Aug 30;13(9):1721. doi: 10.3390/v13091721 (PMC8473162; doi:10.3390/v13091721)
Supplement: Supplementary file 1 [file viruses-13-01721-s001.zip › HCV WG Manuscript_Supplemental Materials - Laboratory Protocol_v1.pdf]

Contents

Specimens 2
Quality Control 2
Laboratory Rooms 2
Equipment and Materials 3
RT-PCR 3
Library Preparation & Sequencing 4
Procedure – RT-PCR 5
Preparation 5
RNA Extraction 5
cDNA Synthesis 5
First Round PCR 7
Second Round PCR 7
Gel Electrophoresis 8
Procedure – Library Preparation and Sequencing 10
Preparation 10
Purification of 2nd PCR Products 10
Amplicon Quantitation and Dilution 11
Nextera XT DNA Sample Preparation 12
PCR Clean-Up/Normalization 14
Normalized Library Quantitation and Dilution 16
Pool Libraries 16
Appendices 18
Appendix 1: Primers 18
WG Amplicon Primers 18
MiDi Amplicon Primers 18
Appendix 2: Reagent Preparation 19
WG Mixes 19
MiDi Mixes 19
15% PEG/2.5M NaCl Stock Solution 20
1X TE Buffer 20
4000 ng/ml Lambda DNA Standard 20
Appendix 3: Thermal Cycler Programs 21
Appendix 4: References 23

Specimens

From a known HCV positive sample:

- Fresh or frozen EDTA, ACD, or heparinized plasma
- Whole blood anti-coagulated with EDTA, ACD, or heparin
- Fresh or frozen serum

Quality Control

Negative Control

- DEPC-treated water

Repeat Sample

- Sample from previous run

Laboratory Rooms

Laboratory rooms used in this procedure are outlined below. In some cases, dependent on individual labs, a single physical room may contain two or more of the room categories below (e.g. Amplification and Sequencing Rooms may be the same room).

	Purpose
**Clean Room**	• Sample receiving<br>• RNA/DNA extractions<br>• Reagent preparation<br>• PCR mix dispensing<br>• Transfer of RNA
**Transfer Room**	• Transfer of cDNA<br>• Transfer of DNA
**Amplification Room**	• Thermocycler runs
**Sequencing Room**	• Purification/Normalization of DNA<br>• DNA quantitation<br>• Preparation of libraries<br>• Sequencing

Equipment and Materials

RT-PCR

<table>
<tr><th colspan="2">Reagents & Storage (Expiry)</th><th>Consumables</th><th>Equipment</th></tr>
<tr><td>RT</td><td>❒ DEPC-treated water, autoclaved, filtered
• Life Technologies, cat. # AM9922</td><td rowspan="3">❒ Sterile, filter, disposable aerosol-safe pipette tips, volume range 10 µl (extra-long tips)

❒ Sterile, filter, disposable aerosol-safe pipette tips, volume range 1 to 1000 µl
---
❒ 0.1 ml Eppendorf disposable aerosol-safe pipette tips for repeater pipette
---
❒ 96 well PCR plates

❒ Micro-caps for 96 well PCR plates

❒ 2 ml screw-cap, polypropylene tubes

❒ Disposable gloves and gowns

❒ Appropriate laboratory disinfectant

❒ Sharps containers

❒ Lint-free tissue

❒ Paper towels or other absorbent paper

❒ Biohazard bags</td><td rowspan="3">❒ Thermocyclers (e.g. MiniAMP Plus)

❒ Multichannel pipette, volume range 0.5 to 10 µl

❒ Multichannel pipette, volume range 20 to 200 µl

❒ Micropipettes, volume range 10 to 1000 µl (designated for DRT)

❒ Centrifuge with plate holders

❒ Microcentrifuge

❒ Vortex

❒ PCR plate supports

❒ Biohazardous waste containers

❒ Refrigerator 2º to 8º C

❒ Freezer -15º to -25º C</td></tr>
<tr><td>-20C</td><td>❒ SuperScript III Reverse Transcriptase (10,000U):
1) SIII Reverse Transcriptase Enzyme (200 U/µl)
2) 0.1 M DTT
• Life Technologies, cat. # 18080-044

❒ RNAseOUT Recombinant Ribonuclease Inhibitor (5,000 U)
• Life Technologies, cat. # 10777-019

❒ Klentaq LA:
1) Klentaq LA DNA Polymerase
2) Klentaq 10X KLA Buffer, pH 9.0
• Cedarlane Laboratories, cat. # 110(DP)

❒ Betaine Solution, 5 M
• Fisher Scientific, cat. # AAJ77507VCR</td></tr>
<tr><td></td><td>❒ Primers, appropriate to amplification target
• See REF_0001 Primer and Tag Sequences for primer ordering info.
• See WI-0010 Sequencing Primers and Preparation of PCR Mixes for mix preparation [to be added to WI-0010 in later version, once method finalized. Until then, it will be included in this SOP]</td></tr>
</table>

Abbreviations: RT: Room Temperature (25°C)
**All items stored at -20°C should be stored in a non-defrosting freezer.

Library Preparation & Sequencing

<table>
<tr><th colspan="2">Reagents & Storage (Expiry)</th><th>Consumables</th><th>Equipment</th></tr>
<tr><td>RT</td><td>❐ DEPC-treated water, autoclaved, filtered
• Life Technologies, cat. # AM9922

❐ Polyethylene glycol 8,000 (500g)
• Fisher Scientific, cat. # AA4344336

❐ 5M Sodium Chloride, Molecular Biology Grade
• Fisher Scientific, cat. # PRV4221

❐ Reagent Alcohol (Ethanol), anhydrous
• Millipore Sigma, cat. # 277649-1L</td><td rowspan="4">❐ Sterile, filter, disposable aerosol-safe pipette tips, volume range 10 µl (extra-long tips)

❐ Sterile, filter, disposable aerosol-safe pipette tips, volume range 1 to 1000 µl
---
❐ 0.1 ml Eppendorf disposable aerosol-safe pipette tips for repeater pipette
---
❐ 96 well PCR plates

❐ Micro-caps for 96 well PCR plates

❐ 2 ml screw-cap, polypropylene tubes

❐ Disposable gloves and gowns

❐ Appropriate laboratory disinfectant

❐ Sharps containers

❐ Lint-free tissue

❐ Paper towels or other absorbent paper

❐ Biohazard bags</td><td rowspan="4">❐ Thermocyclers (e.g. MiniAMP Plus)

❐ DynaMag 96 Side Magnet
• Life Technologies, cat.# 12-331-D

❐ DynaMag 2 Magnet
• Life Technologies, cat.# 12-321-D

❐ Multichannel pipette, volume range 0.5 to 10 µl

❐ Multichannel pipette, volume range 20 to 200 µl

❐ Micropipettes, volume range 10 to 1000 µl (designated for DRT)

❐ Centrifuge with plate holders

❐ Microcentrifuge

❐ Vortex

❐ PCR plate supports

❐ Biohazardous waste containers

❐ Refrigerator 2º to 8º C

❐ Freezer -15º to -25º C</td></tr>
<tr><td rowspan="2">-20C</td><td>❐ Nextera XT Index Kit SetA
• Illumina, cat. # FC-131-2001

❐ Nextera XT Index Kit
• Illumina, cat. # FC-131-1001

❐ Nextera XT DNA Library Preparation Kit
• Box 1:
  • Amplicon Tagment Mix (ATM)
  • Tagment DNA Buffer (TD)
  • Nextera PCR Master Mix (NPM)
• Illumina, cat. # FC-131-1096</td></tr>
<tr><td>❐ Primers, appropriate to amplification target
• See REF_0001 Primer and Tag Sequences for primer ordering info.
• See WI-0010 Sequencing Primers and Preparation of PCR Mixes for mix preparation [to be added to WI-0010 in later version, once method finalized. Until then, it will be included in this SOP]</td></tr>
<tr><td>4C</td><td>❐ AMPure XP beads
• Life Technologies, cat. # A63880

❐ Nextera XT DNA Library Preparation Kit
• Box 2
  • Neutralize Tagment Buffer (NT)
• Illumina, cat. # FC-131-1096</td></tr>
</table>

Abbreviations: RT: Room Temperature (25°C)
**All items stored at -20°C should be stored in a non-defrosting freezer.

Procedure – RT-PCR

Preparation

The following components of the assay are outlined in the respective Appendix sections at the end of the document:

- Primer information
- Reagent preparation protocols (e.g. RT-PCR mix recipes)
- Thermal Cycler programs

RNA Extraction

RNA should be extracted according to manufacturer protocol using a BioMerieux easyMAG platform, or a suitable alternative.

cDNA Synthesis

Procedural Note

- All pipette tips used in this procedure should be filtered, sterile and aerosol-safe.
- Clean and disinfect the pipettes and workbench before and after use.
- Depending on the number of samples to be processed, use a 96-well PCR plate or 0.2 ml PCR strip-tubes. Instructions below will refer only to plates for clarity.
- Both the WG and MiDi amplicons are processed separately from one another (in separate plate, separate Nextera reactions), and only combined immediately prior to MiSeq sequencing (at the library pooling step).

1. Switch on a thermocycler in the Amplification Room if not already on.

Steps 2-8 should be carried out in the *Clean Room.*

2. Label two 96-well plates with the plate ID, date, and initials: one for **HCV WG**, one for **HCV miDi**.

3. Thaw one tube each of **HCV WG "RT1"** and **miDi "RT1" mixes**. If there is leftover mix, label the tube with your initials and amount used before returning to freezer.

NOTE: Once thawed, the PCR mix should be used immediately or kept at 4°C for up to 1 hr.

4. Remove RNA extracts (patients and controls) from 4°C or -20°C.

5. Gently vortex the "RT1" mix and spin briefly. Transfer **8 µl** of mix into alternating columns of a 96-well plate. Loosely cover with strip-caps.

6. Spin down RNA extract tubes using the picofuge with tube-strip adapter, if necessary. Carefully open the lids using lint-free tissue.

7. Uncovering one column of the plate at a time, transfer **5 µl** of RNA extract into each well containing "RT1" mix (total reaction volume = 13uL).

8. When finished with a column, replace the strip-cap and apply firm pressure with the cap sealer. Proceed to the next column(s) as above until finished.

9. Thaw one tube each of **HCV WG "RT2"** and **miDi "RT2" mixes**. Gently vortex and spin briefly. Aliquot 7 µl per reaction + extra to be added to the "RT1" plates. If there is leftover mix, label the tube with your initials and amount used before returning to freezer.

NOTE: Once thawed, the PCR mix should be used immediately or kept at 4°C for up to 1 hr.

10. Take the RT1 plates and aliquoted "RT2" mix to the Transfer Room. If liquid remains on the sides of the wells, centrifuge for 1 min using the benchtop 96-well plate centrifuge.

11. Place each of the RT1 plates into a thermocycler in the Amplification Room, close and tighten the lid, and start the program as follows:
    a. Press <*Set Up Run*> then <*Open Method*>.
    b. Select folder <*HCV*>.
    c. Select program method < *HCV WG, miDi rt1*> (see WI-0008, Thermocycler Methods).
    d. Check that the volume is at least 13 µl. If not, press <*Edit*> and change volume.
    e. Press <Next> then <*Start Run*>. The thermocycler will begin cycling as soon as it reaches temperature. Total duration: 5min

12. Do not wait to ramp down to 4°C! Proceed to next immediately.

13. Take a container of flaked ice to the Transfer Room. **IMMEDIATELY** after the 5 min @ 65°C has completed, place the completed "RT1" denatured plate on ice for 1 min.

14. Using lint-free tissue, uncover one column of the plate at a time. Transfer **7 µl** of "RT2" mix into each well containing denatured RNA (total reaction volume = 20uL).

15. When finished with a column, cover with a fresh strip-cap and apply firm pressure with the cap sealer. Proceed to the next column(s) as above until finished.

16. If liquid remains on the sides of the wells, centrifuge for 1 min at 161 RCF (1000 rpm) using the benchtop 96-well plate centrifuge.

17. Place each of the RT plates into a thermocycler the Amplification Room, close and tighten the lid, and start the program as follows:
    a. Press <*Set Up Run*> then <*Open Method*>.
    b. Select folder <*HCV*>.
    c. Select program method < *HCV WG rt2* > *or* < *HCV miDi rt2*> (see WI-0008, Thermocycler Methods).
    d. Check that the volume is at least 20 µl. If not, press <*Edit*> and change volume.

e. Press <Next> then <*Start Run*>. The thermocycler will begin cycling as soon as it reaches temperature. Total duration: ~1hr (miDi) and ~2.5hr (WG)

First Round PCR

1. *In the Clean Room,* thaw one tube each of **HCV WG 1st Round** and **miDi 1st Round PCR mixes**. Gently vortex and spin briefly. Aliquot 20 µl per reaction + extra to be added to the cDNA plate. If there is leftover mix, label the tube with your initials and amount used before returning to freezer.

NOTE: Once thawed, the PCR mix should be used immediately or kept at 4°C for up to 1 hr.

2. *In the Transfer Room,* uncover one column of the cDNA plate at a time using lint-free tissue. Transfer **20 µl** of 1st Round PCR mix into each well containing cDNA (total reaction volume = 40uL).

3. When finished with a column, cover with a fresh strip-cap and apply firm pressure with the cap sealer. Proceed to the next column(s) as above until finished.

4. Gently tap the plate support to remove any bubbles from the bottom of the plate.

5. Place each of the PCR plates into a thermocycler in the Amplification Room, close and tighten the lid, and start the program as follows:
   a. Press <*Set Up Run*> then <*Open Method*>.
   b. Select folder <*HCV*>.
   c. Select program method < *HCV WG 1st and 2nd* > *or* < *HCV miDi 1st and 2nd* > (see WI-0008, Thermocycler Methods).
   d. Check that the volume is at least 40 µl. If not, press <*Edit*> and change volume.
   e. Press <Next> then <*Start Run*>. The thermocycler will begin cycling as soon as it reaches temperature. Total duration: ~3h40m (miDi) and ~6h40m (WG)

6. Once finished, proceed with 2nd Round PCR or store the 1st Round PCR products at RT° or 4°C for later processing.

Second Round PCR

NOTE: All pipette tips used in this procedure should be aerosol-safe. Clean and disinfect the pipettes and workbench before and after use.

1. Switch on a thermocycler in the Amplification Room if not already on.

Steps 2-4 should be carried out in the Clean Room.

2. Label a 96-well plate with the plate ID, date, and initials.

3. Thaw one tube each of **HCV WG 2nd Round** and **miDi 2nd Round PCR mixes**. If there is leftover mix, label the tube with your initials and amount used before returning to freezer.

NOTE: Once thawed, the PCR mix should be used immediately or kept at 4°C for up to 1 hr.

4. Gently vortex the 2nd Round PCR mix and spin briefly. Transfer **18 µl** of mix into each well.

5. Loosely cap the completed columns. Take the plates containing 2nd Round PCR mix and 1st Round PCR products to the Transfer Room for transfer.

6. Working with one column at a time, remove and discard the strip-caps from the 1st Round PCR plate, using lint-free tissue to avoid contamination by aerosols.

7. Transfer **2 µl** of 1st Round PCR product into the corresponding wells containing 2nd Round PCR mix (total reaction volume = 20uL)..

8. Apply fresh strip-caps to seal the completed columns of both 1st and 2nd Round PCR plates. Proceed to the next column(s) as above until finished.

9. Gently tap the plate to remove any bubbles from the bottom of the wells.

10. Place each of the PCR plates into a thermocycler the Amplification Room, close and tighten the lid, and start the program as follows:
    a. Press <*Set Up Run*> then <*Open Method*>.
    b. Select folder <*HCV*>.
    c. Select program method < *HCV WG 1st and 2nd* > *or* < *HCV miDi 1st and 2nd* > (see WI-0008, Thermocycler Methods).
    d. Check that the volume is at least 20 µl. If not, press <*Edit*> and change volume.
    e. Press <Next> then <*Start Run*>. The thermocycler will begin cycling as soon as it reaches temperature. Total duration: ~3h40m (miDi) and ~6h40m (WG)

11. Once finished, proceed to the next step or store the 2nd Round PCR products at 4°C in the Sequencing Room for later processing.

Gel Electrophoresis

Both the MiDi and WG amplicons should be visualized on a 1% agarose gel to confirm amplification.

1. NOTE: All PCR mixes for this procedure are colourless. Be careful when loading 2nd Round PCR products into the agarose gel.

2. Once finished, proceed to the next step or store the 2nd Round PCR products at 4°C in the Sequencing Room for later processing.

Expected results

Negative Control

- Should have no specific bands appearing on the gel. A negative control that tests positive invalidates the run, and all samples must be re-extracted.

Samples

- Should have one band at the appropriate distance compared to the ladder(s).
    - HCV WG 2nd Round PCR product is ~8.4 kb.
    - HCV miDi 2nd PCR product is ~1.2 kb.
    - A repeat sample that tests negative does not invalidate the run.

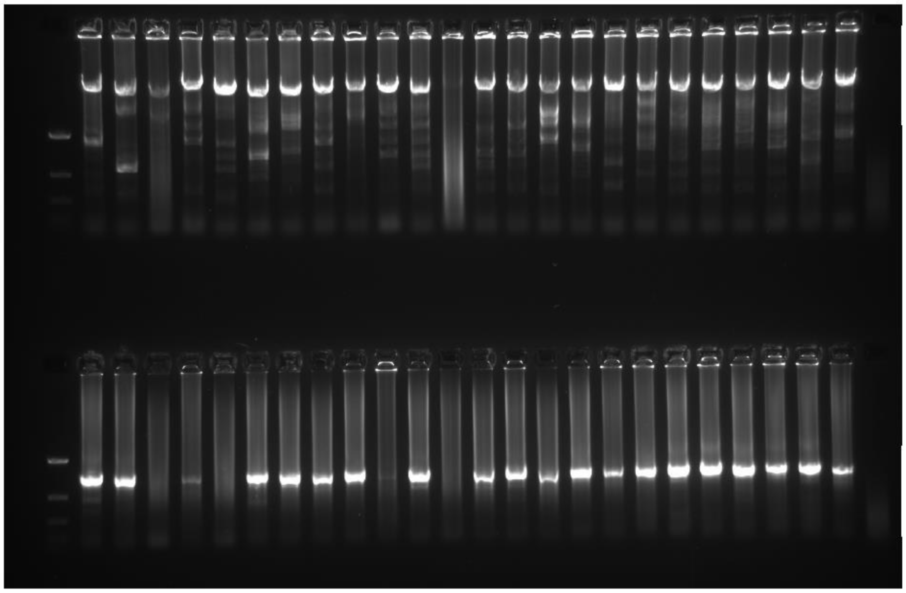

HCV WG1 ~ 9000 bp
HCV WG2 ~9000 bp

HCV miDi/NS5A ~ 1260 bp

Procedure – Library Preparation and Sequencing

Preparation

Procedural note

- The WG and MiDi amplicons are to be processed by Nextera separately, as per protocol. Both amplicons will be pooled when preparing the final DNA library for loading onto the MiSeq.
- The WG and MiDi amplicons for the same samples should be tagged using different Illumina indices.
    - The use of different tags allows for comparing the overlapping region of both amplicon from each sample.
    - Note: the user could tag amplicons from the same sample with the same indices to simplify lab work, sample sheet creation and avoid generating two data sets per sample.

Purification of 2nd PCR Products

1. Prepare 70% ethanol fresh daily, e.g. 7 ml of 100% ethanol + 3 ml DEPC water (adjust volume based on number of samples). Mix well.

2. Vortex AMPure XP beads for 20 sec to completely re-suspend. Dilute beads 1:3 in 15% PEG/2.5M NaCl solution, e.g. 950 µl of AMPure XP + 1900 µl of 15% PEG/2.5M NaCl (adjust volume based on number of samples). Mix well by vortexing.

3. Label a 96-well PCR plate. (Note: depending on the number of samples to be processed, a single plate can be used for Purification, Elution, and Dilution.)

4. Add 7.5 µl of DEPC water to sample wells according to the 2nd PCR layout. Pipette 7.5 µl of 2nd PCR product and mix well by pipetting up and down 5 times. Change tips between samples.

5. Add 27 µl of (1:3) AMPure XP Beads to each sample well containing the diluted PCR product with a multichannel pipette. Cap the plate, vortex to mix and briefly centrifuge at 1000 g.

6. Incubate for 10 min at room temperature on the plate mixer at 1800 rpm. After incubation, spin briefly.

7. Place the PCR plate on the Low Volume 24-Post Magnet Plate and incubate for 5 min at room temperature.

8. With the plate still situated on the Magnet Plate, use a multichannel pipette to carefully remove and discard the supernatant without disturbing the separated magnetic beads. Change tips between samples.

9. With the plate still situated on the Magnet Plate, add 200 µl of freshly prepared 70% ethanol (from Step 1) to each sample well. Incubate for 1 min at room temperature.

10. With the plate still situated on the Magnet Plate, carefully remove and discard the supernatant without disturbing the separated magnetic beads. Change tips between samples.

11. Repeat Steps 9-10. Remove as much of the supernatant as possible.

12. With the plate still situated on the Magnet Plate, incubate for 3-5 min at room temperature uncapped. Do not over-dry.

13. With the plate still situated on the Magnet Plate, add 40 µl of DEPC water to each sample well. Remove from the magnet to a plate base and cap the wells.

14. Move the plate around on the magnet posts to mobilize the beads, then briefly vortex and centrifuge at 1000 g. *Do not vortex excessively or the beads may break!*

15. Incubate for 2 min on the bench at room temperature, then return the plate to the Magnet Plate and incubate for another 2 min.

16. With the plate still situated on the Magnet Plate, carefully transfer 20 µl of supernatant to a new labelled 96-well PCR plate (or clean wells of the same plate) without disturbing the separated magnetic beads.

17. The purified amplicon libraries can be stored at -15° to -25°C until ready to proceed to the quantitation step.

Amplicon Quantitation and Dilution

1. Users should quant each individual amplicon's DNA concentration using a suitable lab instrument (e.g. Qubit fluorometer). Protocol not included here.

2. For each sample, calculate the volume (µl) of DEPC water needed for diluting 3 µl of sample to 1.0 ng/µl using the following formula:

$$DEPC\ water\ volume\ (uL) = \frac{3 \times Conc.\,(\frac{ng}{uL})}{1.0} - 3$$

3. Label a new 96-well PCR plate (or clean wells of the same plate) for sample dilution to 1.0 ng/µl.

4. Following the calculations in Step 3, pipette the appropriate amount of DEPC water needed for each sample into the wells of the dilution plate, according to the PCR layout.

5. Using a multichannel pipette, add 3 µl of sample into the dilution plate according to the PCR layout. Cap the plate, then vortex and centrifuge briefly. Amplicons can be stored at -15° to -25°C until ready to proceed to Nextera XT.

Nextera XT DNA Sample Preparation

Preparations

In the Sequencing Room, prepare for **Tagmentation** and **Neutralize Tagmentation**

1. Remove **ATM** (Amplicon Tagment Mix), **TD** (Tagment DNA Buffer), and input DNA (1ng/uL) plate from -15° to -25°C storage and thaw on ice.

2. After thawing, ensure all reagents are adequately mixed by gently inverting the tubes 3–5 times, followed by a brief spin in a microcentrifuge.

3. Label a new 96-well PCR plate **Tagmentation**.

4. Mix ATM (2 µl per sample) and TD (4 µl per sample) together and aliquot 6 µl into each sample well of the Tagmentation plate.

5. Cover the Tagmentation plate with caps and store on ice.

6. If needed, prepare a plate of **NT** (Neutralize Tagment Buffer). Remove NT tube(s) from 4°C. Visually inspect NT for precipitates. If precipitates are present, vortex until all particulates are re-suspended, followed by a brief spin. Aliquot 50 µl into each well of a clean, labelled 96-well PCR plate.

7. Ensure that NT plate is at room temperature and free of precipitates before use; 2 µl of NT is needed per sample.

8. Take 1.0 ng/µl dilution, Tagmentation, and NT plates to the Transfer Room for transfer.

In the Clean Room, prepare for **PCR Amplification Adding Indices**

9. Remove **NPM** (Nextera PCR Master Mix) and the index primers (i5 and i7) from -15° to -25°C storage and thaw on the bench at room temperature. Record the indices used in order to create a MiSeq Sample Sheet later or refer to an existing Sample Sheet.

10. After thawing, gently invert each tube 3–5 times to mix and briefly centrifuge the tubes in a microcentrifuge. Spin indexes very well to avoid contamination when opening the tubes.

11. Label a new 96-well PCR plate **NTA** (Nextera XT Tagment Amplicon Plate).

12. Combine NPM (4.5 µl per sample) and index 1 (1.5 µl per sample) in 0.2 ml strip-tubes. Mix well and spin briefly. Using a multichannel pipette, aliquot 6 µl to the bottom of each sample well of the **NTA** plate.

13. Using a multichannel pipette add 1.5 µl of index 2 primers to the bottom of each well of the **NTA** plate. ***Changing tips between wells is required to avoid cross-contamination.***

14. Cover the plate with film and seal tightly. Store on ice and take to the Transfer Room for transfer.

Tagmentation

NOTE: The reaction does not need to be assembled on ice.

1. Wrap all four plates (1.0 ng/µl DNA dilution, Tagmentation, NT, NTA) in large Kimwipes and centrifuge briefly (at 1000 g) in the benchtop 96-well plate centrifuge in the Sequencing Room.

2. Add 2 µl of input DNA at 1.0 ng/µl (2.0 ng total) to each sample well of the **Tagmentation** plate using a multichannel pipette. Gently pipette up and down 5 times to mix. Change tips between samples.

3. Cover the **Tagmentation** plate with caps and centrifuge briefly (at 280 g) in the benchtop 96-well plate centrifuge in the Sequencing Room.

4. Place the **Tagmentation** plate in a thermocycler in the Amplification Room. Close, tighten the lid and start the program as follows:
    a. Press <*Set Up Run*> then <*Open Method*>.
    b. Select folder <*MiSeq*>.
    c. Select program method < *tagmentation* > (see WI-0008, Thermocycler Methods).
    d. Check that the volume is at least 8 µl. If not, press <*Edit*> and change volume.
    e. Press <Next> then <*Start Run*>. The thermocycler will begin cycling as soon as it reaches temperature. Total duration: ~3.5min

5. Once the sample reaches 10°C proceed immediately to <u>Neutralize Tagmentation.</u>

Neutralize Tagmentation

6. Carefully remove the caps and add 2 µl of **NT** Buffer to each well of the **Tagmentation** plate. Gently pipette up and down 5 times to mix. Change tips between samples.

7. Cover the **Tagmentation** plate with film and seal tightly. Centrifuge briefly (at 280 g) in the benchtop 96-well plate centrifuge in the Sequencing Room.

8. Return **Tagmentation** plate to the Transfer Room and incubate at room temperature for 5 minutes.

PCR Amplification Adding Indices

9. Using a multichannel pipette, add 7.5 µl of **Tagmentation** to each well of the **NTA** plate containing the **NPM** and indices. Gently pipette up and down 5 times to mix. Change tips between samples to avoid index and sample cross contamination.

10. Cover the NTA plate with caps and centrifuge briefly (at 280 g) in the benchtop 96-well plate centrifgue in the Sequencing Room. The Tagmentation plate can be discarded.

11. Place the NTA plate in a thermocycler in the Amplification Room. Close, tighten the lid and start the program as follows:
    a. Press <*Set Up Run*> then <*Open Method*>.
    b. Select folder <*MiSeq*>.
    c. Select program method < *add indices* > (see WI-0008, Thermocycler Methods).
    d. Check that the volume is at least 15 µl. If not, press <*Edit*> and change volume.
    e. Press <Next> then <*Start Run*>. The thermocycler will begin cycling as soon as it reaches temperature. Total duration: ~35min

12. Once finished, proceed to the next step or store the Nextera products at 4°C in the Sequencing Room for later processing. The plate can remain on the thermocycler overnight.

PCR Clean-Up/Normalization

All steps below should be performed in the Sequencing Room.

NOTE: To use the Nimbus for semi-automated normalization, see WI-R0001, MiSeq Sample Preparation Using the NIMBUS96.

1. Prepare 70% ethanol fresh daily, e.g. 7 ml of 100% ethanol + 3 ml DEPC water (adjust volume based on number of samples). Mix well.

2. Vortex AMPure XP beads for 20 sec to completely re-suspend. Prepare 1:50 AMPure XP beads in 15% PEG/2.5M NaCl solution, e.g. 20 µl of AMPure XP + 980 µl of 15% PEG/2.5M NaCl (adjust volume based on number of samples). Mix well by vortexing.

3. Label a clean 96-well PCR plate. Pour the re-suspended 1:50 AMPure XP beads solution into a clean reagent reservoir. Using a p10 multichannel pipette, add 9 µl of diluted beads to each sample well according to the PCR layout.

4. Vortex briefly and centrifuge the NTA plate at 1000 g for 1 min. Transfer 5 µl of NTA product to the appropriate well containing 9 µl diluted beads.

5. Cap the NTA and the normalization plates. Vortex the normalization plate to mix well, then centrifuge at 1000 g for 1 min. Incubate for 10 min. at room temperature on the plate mixer at 1800 rpm.

6. Pool the content of all wells containing the same amplicon. Normalize each project (PR-RT, INT, HCV Whole Genome, etc.) separately.

7. Pipette the pooled well(s) to the bottom of a labelled Eppendorf DNA LoBind 1.5 ml tube (one tube per project). Vortex to mix and spin briefly.

8. Place the tubes in a Magnetic Particle Collector (MPC) and incubate for 5 min at room temperature. NOTE: Beads may not be visible at this stage; they will become visible during the ethanol wash.

9. With the tubes still in the MPC, carefully remove the supernatant and discard, without disturbing the beads.

10. Add 150 µl of fresh 70% ethanol into a sample well (or column of sample wells) of the normalization plate. Pipette up and down a couple of times, then transfer to the next well. Continue until all wells are washed.

11. Pool the 70% ethanol from the last sample well/column into the appropriate 1.5 ml tube containing samples/beads. Incubate for 1 min on the MPC, turning the tubes 180° every 30 sec.

12. With the tubes still in the MPC, carefully remove the supernatant and discard, without disturbing the beads.

13. Add 1 ml of fresh 70% ethanol into the 1.5 ml tubes. Incubate for 1 min on the MPC, turning the tubes 180° every 30 sec.

14. With the tubes still in the MPC, carefully remove and discard the supernatant without disturbing the beads.

15. Repeat Steps 13-14 once.

16. Cap the tubes and spin briefly to bring the remaining liquid down. Return the tubes to the MPC and incubate for 1 min, then carefully remove and discard the rest of the supernatant.

17. To remove the last traces of ethanol, incubate the tubes at room temperature for about 5 min. *with the lids open.* Do not over-dry.

18. Elute the beads with ~1 µl of DEPC water per sample, e.g. for 96 samples, add 100 µl of DEPC water to the tubes. Exception: if ≤10 samples, elute with 15 µl.

19. Cap and vortex until the pellet is completely re-suspended. <u>*Do not vortex excessively or the beads may break!*</u>

20. Spin the tubes briefly. Incubate on the bench for 2 min, then return the tubes to the MPC and incubate for another 2 min.

21. With the tubes still in the MPC, carefully transfer the supernatant to clean, labelled 1.5 ml tubes. Leave 5-10 µl behind to avoid pipetting beads.

22. The normalized libraries can be stored at -15° to -25°C until ready to proceed to the quantitation step. Tubes with beads should be stored at 2° to 8°C.

Normalized Library Quantitation and Dilution

Quantitation

Quantitation, dilution, and pooling libraries may be performed up to 1 week prior to MiSeq sequencing setup.

a. Users should quant each individual library's DNA concentration using a suitable lab instrument (e.g. Qubit fluorometer). Protocol not included here.
   - Libraries should be quanted in duplicate
   - Use a 0.5-10 µl pipette with long 10 µl tips for libraries in 1.5 ml tubes.
   - Check that the duplicate samples have consistent/similar concentrations. The difference between measurements must be ≤10% of the mean; otherwise, repeat the quant.
   - Samples below 5ng/uL should be repeated

Dilution

1. From the original library concentrations, take the average of the two readings (in ng/uL).

2. Based on the average concentration results, calculate the volume (µl) of DEPC water needed for diluting 3 µl of the library to **1.3 ng/µl**:

$$DEPC\ water\ volume\ (uL) = \frac{3 \times Conc.\left(\frac{ng}{uL}\right)}{1.3} - 3$$

Pool Libraries

Procedural Note

To achieve a minimum coverage of 1000 reads, MiSeq sequencing can accommodate the equivalent of 48 samples x 9000 bp. In the context of this HCV sequencing assay, which includes the WG and MiDi amplicons, 42 samples can be sequenced in parallel to target this 1000x coverage across the HCV genome. Users may adjust samples numbers to achieve greater genome coverage (by decreasing sample numbers) or to increase sample throughput (thereby decreasing expected read coverage).

All steps below should be performed in the Sequencing Room.

Gather Libraries and Pool

1. Gather the purified, normalized, diluted, tagged DNA libraries for both the WG and MiDi libraries.

2. Pool the WG and MiDi libraries in a 1:0.14 ratio, respectively, to achieve a minimum total volume of 10uL.
    - In most cases dependent on samples being processed, users should have sufficient library to pool 8.77uL of WG library with 1.23uL of MiDi library.

3. Mix well by vortexing then centrifuge briefly. Keep on ice, or store library at -15° to -25°C until ready to proceed to the next step.

4. Proceed to MiSeq sequencing using a 2x250bp kit, according to manufacturer protocol.

Appendices

Appendix 1: Primers

WG Amplicon Primers

Primer	Reaction	Sequence (5’ to 3’)	H77 coordinates	Directionality
oligo dA20	RT	AAAAAAAAAAAAAAAAAAAA	9418-9437	R
Pr3	RT	GGCGGAATTCCTGGTCATAGCCTCCGTGAA	8616–8645	R
1abGENF1bp	PCR1	GGGTCGCGAAAGGCCTTGTGGTACTGCC	266-293	F
TIM-Pr3	PCR1	CAGGAAACAGCTATGACGGCGGAATTCCTGGTCATAG CCTCCGTGAA	8616–8645	R
1abGENF2	PCR2	GTACTGCCTGATAGGGTGCTTGCGAGTGCC	286-315	F
Pr6	PCR2	AATTCCTGGTCATAGCCTCCGTGAAGACTC	8611–8640	R

MiDi Amplicon Primers

Primer	Reaction	Sequence (5’ to 3’)	H77 coordinates	Directionality
oligo dA20	RT	AAAAAAAAAAAAAAAAAAAA	9418-9437	R
Pr1	PCR1	TGGGGTTCGCGTATGATACCCGCTGCTTTGA	8245–8275	F
Pr2	PCR1	TGGGGTTTTCTTACGACACCAGGTGCTTTGA	8245–8275	F
oligo dA20-TIM	PCR1	CAGGAAACAGCTATGACAAAAAAAAAAAAAAAAAAAA	9418-9437	R
Pr4	PCR2	CCGTATGATACCCGCTGCTTTGACTCAAC	8253–8281	F
Pr5	PCR2	TCCTACGACACCAGGTGCTTTGATTCAAC	8253–8281	F
TIM	PCR2	CAGGAAACAGCTATGAC		R

Abbreviations: RT: Reverse transcription; PCR1: 1st Round PCR; PCR2: Nested PCR

Appendix 2: Reagent Preparation

WG Mixes

RT1 mix

Component	1X
DEPC-treated water	5.52
Primer **oligo dA20**, 25uM	2
dNTP, 25mM	0.4
Primer **Pr3**, 25uM	0.08
Subtotal	8uL
RNA extract	5uL

RT2 mix

Component	1X
DEPC-treated water	2
Klentaq 10X KLA Buffer, pH 9.0	1
0.1M DTT	1
RNAseOUT (40U/ul)	1
SuperScript III RT Enzyme (200U/ul)	2
Subtotal	7uL
RT1 template	13uL

1st Round PCR mix

Component	1X
DEPC-treated water	1.88
Klentaq 10X KLA Buffer, pH 9.0	4
Betaine, 5M	12
dNTP, 25mM	0.32
Primer **1abGENF1bp**, 25uM	0.64
Primer **TIM-Pr3**, 25uM	0.64
Klentaq LA enzyme	0.52
Subtotal	20uL
cDNA template	20uL

2nd Round PCR mix

Component	1X
DEPC-treated water	8.94
Klentaq 10X KLA Buffer, pH 9.0	2
Betaine, 5M	6
dNTP, 25mM	0.16
Primer **1abGENF2**, 25uM	0.32
Primer **Pr6**, 25uM	0.32
Klentaq LA enzyme	0.26
Subtotal	18uL
1st Round PCR template	2uL

MiDi Mixes

RT1 mix

Component	1X
DEPC-treated water	5.6
Primer **oligo dA20, 25uM**	2
dNTP, 25mM	0.4
Subtotal	8uL
RNA extract	5uL

RT2 mix

Component	1X
DEPC-treated water	3
Klentaq 10X KLA Buffer, pH 9.0	1
0.1M DTT	1
RNAseOUT (40U/ul)	1
SuperScript III RT Enzyme (200U/ul)	1
Subtotal	7uL
RT1 template	13uL

1st Round PCR mix

Component	1X
DEPC-treated water	2.2
Klentaq 10X KLA Buffer, pH 9.0	4
Betaine, 5M	12
dNTP, 25mM	0.32
Primer **Pr1+Pr2 (1:1),** 25uM*	0.64
Primer **oligo dA20-TIM,** 25uM	0.64
Klentaq LA enzyme	0.2
Subtotal	20uL
cDNA template	20uL

2nd Round PCR mix

Component	1X
DEPC-treated water	9.1
Klentaq 10X KLA Buffer, pH 9.0	2
Betaine, 5M	6
dNTP, 25mM	0.16
Primer **Pr4+Pr5 (3:4)**, 25uM**	0.32
Primer **TIM**, 25uM	0.32
Klentaq LA enzyme	0.1
Subtotal	18uL
1st Round PCR template	2uL

*Users can create a 25uM working solution of Pr1 and Pr2 primers at a 1:1 ratio by combining both 25uM primers in equal volume.
**Users can create a 25uM working solution of Pr4 and Pr5 primers at a 3:4 ratio by combining 30uL Pr4 (25uM) + 40uL Pr5 (25uM). Adjust volumes as needed, keeping the proportion of each primer the same.

15% PEG/2.5M NaCl Stock Solution

1. To make 30% PEG-8000 stock: measure 75 g of PEG-8000 and 100 ml DEPC water in a 500 ml bottle.
2. Invert to mix, then top up to 250 ml with DEPC water.
3. Measure 250 ml of 5M NaCl solution and mix with the 250 ml 30% PEG-8000.
4. Filter with 0.2 µM filter unit under vacuum.
5. Store at 4°C for up to 7 years.

1X TE Buffer

1. Make a 1:20 dilution of 20X TE Buffer with DEPC water, e.g. 25 ml 20X TE + 475 ml water.
2. Store at 4°C for up to 1 year.

4000 ng/ml Lambda DNA Standard

1. Make a 1:25 dilution of the Lambda DNA Standard (100 µg/ml) in a 1.5 ml microcentrifuge tube using "clean" 1X TE Buffer made with DEPC water, e.g. 40 µl Lambda + 960 µl 1X TE.
2. Vortex for 10 sec. to mix well.
3. Store at 4°C for up to 6 months.

Appendix 3: Thermal Cycler Programs

RNA denaturation ("HCV WG, miDi rt1")

Temperature	Length	Cycles	Adjustments
65°C	5min	1	
4°C	∞*		

**Do not wait to ramp down to 4°C! Proceed to next step immediately*

cDNA synthesis – WG amplicon ("HCV WG rt2")

Temperature	Length	Cycles	Adjustments
25°C	10min	1	
42°C	30min	1	
45°C	30min	1	
50°C	30min	1	
55°C	30min	1	
70°C	15min	1	
10°C	∞		

cDNA synthesis – MiDi amplicon ("HCV miDi rt2")

Temperature	Length	Cycles	Adjustments
25°C	10min	1	
42°C	10min	1	
45°C	10min	1	
50°C	10min	1	
55°C	10min	1	
85°C	5min	1	
10°C	∞		

cDNA synthesis -WG amplicon ("HCV WG 1st and 2nd")

Temperature	Length	Cycles	Adjustments
94°C	2min	1	
94°C<br>68°C*<br>68°C	15sec<br>20sec<br>12min	} 30	*-0.5°C/cycle (start on 1st cycle)
4°C	∞		

1st and 2nd Round PCR -MiDi amplicon ("HCV miDi 1st and 2nd")

Temperature	Length	Cycles	Adjustments
94°C	2min	1	
94°C<br>60°C*<br>68°C	15sec<br>20sec<br>4min	} 30	*-0.5°C/cycle (start on 1st cycle)
94°C<br>60°C*<br>68°C	15sec<br>20sec<br>4min	} 10	*-0.5°C/cycle (start on 1st cycle)
4°C	∞		

Tagmentation ("tagmentation")

Temperature	Length	Cycles	Adjustments
55°C	3min	1	
10°C	∞		

Tagging ("add indices")

Temperature	Length	Cycles	Adjustments
72°C	3min	1	
95°C	30sec	1	
95°C<br>55°C*<br>72°C	10sec<br>30sec<br>30sec	} 12	
72°C	5min	1	
10°C	∞		

Appendix 4: References

1. Zhang EZ, Bartels DJ, Frantz JD, Seepersaud S, Lippke JA, Shames B, Zhou Y, Lin C, Kwong A, and Kieffer TL. Development of a sensitive RT-PCR method for amplifying and sequencing near full-length HCV genotype 1 RNA from patient samples. Virology Journal 2013, 10:53.
2. Nextera XT DNA Library Preparation Guide. Illumina Proprietary, PN 15031942 Rev. E. January 2015.
3. Agencourt AMPure XP PCR Purification – Instructions for Use. Beckman Coulter, PN B37419AA. August 2013.
