# Supplementary material for: Validation of a Genotype-Independent Hepatitis C Virus Near-Whole Genome Sequencing Assay
Source: Viruses. 2021 Aug 30;13(9):1721. doi: 10.3390/v13091721 (PMC8473162; doi:10.3390/v13091721)
Supplement: Supplementary file 1 [file viruses-13-01721-s001.zip › HCV WG Manuscript_v7_MDPPI_Viruses_Suppl_FigsTable.pdf]

Supplemental Materials

Table S1. Primers used in the genotype-independent sequencing protocol. Primers were purified by standard desalting.

STEP	AMPLICON	NAME	SEQUENCE (5' TO 3')	DIRECTION
**RT**	WG	oligo dA20	AAAAAAAAAAAAAAAAAAAA	Reverse
**RT**	WG	Pr3	GGCGGAATTCCTGGTCATAGCCTCCGTGAA	Reverse
**PCR**	WG	1abGENF1bp	GGGTCGCGAAAGGCCTTGTGGTACTGCC	Forward
**PCR**	WG	TIM-Pr3	CAGGAAACAGCTATGACGGCGGAATTCCTGGTCATAGCCTCCGTGAA	Reverse
**NESTED PCR**	WG	1abGENF2	GTACTGCCTGATAGGGTGCTTGCGAGTGCC	Forward
**NESTED PCR**	WG	Pr6	AATTCCTGGTCATAGCCTCCGTGAAGACTC	Reverse
**PCR**	MiDi	Pr1	TGGGGTTCGCGTATGATACCCGCTGCTTTGA	Forward
**PCR**	MiDi	Pr2	TGGGGTTTTCTTACGACACCAGGTGCTTTGA	Forward
**PCR**	MiDi	oligo dA20-TIM	CAGGAAACAGCTATGACAAAAAAAAAAAAAAAAAAAA	Reverse
**NESTED PCR**	MiDi	Pr4	CCGTATGATACCCGCTGCTTTGACTCAAC	Forward
**NESTED PCR**	MiDi	Pr5	TCCTACGACACCAGGTGCTTTGATTCAAC	Forward
**NESTED PCR**	MiDi	TIM	CAGGAAACAGCTATGAC	Reverse

Table S2. FDA guideline of Reference Strains for Reporting of Amino Acid Sequence Data.

GENOTYPE	REFERENCE STRAIN	GENBANK ACCESSION ID	LENGTH (BP)	NUCLEOTIDE POSITIONS NS3-4A	NUCLEOTIDE POSITIONS NS5A	NUCLEOTIDE POSITIONS NS5B
**GENOTYPE 1A**	H77	NC_004102	9646	3420-5474	6258-7601	7602-9377
**GENOTYPE 1B**	Con1	AJ238799	9030	3420-5474	6258-7598	7599-9374
**GENOTYPE 2**	JFH-1	AB047639	678	3431-5485	6269-7666	7667-9442
**GENOTYPE 3**	S52	GU814263	555	3436-5490	6274-7629	7630-9402
**GENOTYPE 4**	ED43	GU814265	497	3419-5473	6257-7591	7592-9364
**GENOTYPE 5**	SA13	AF064490	408	3328-5382	6166-7515	7516-9291
**GENOTYPE 6**	EUHK2	Y12083	340	3374-5428	6212-7564	7565-9340

Table S3. HCV resistance-associated substitutions (RAS) in genotype 1a and 1b that were analyzed for accuracy. X indicates a variant with any mutation except the wildtype amino acid residue.

NS3	NS5a	NS5b
V36A/G/L/M/I	M28T/V/A (GT1a only)	L159F
T54A/C/G/S	L28T/V/A (GT1b only)	S282T
V55A/I	Q30E/H/R/G/K/L/D (GT1a only)	L320F
Y56H	R30E/H/G/K/L/D (GT1b only)	
Q80K/R	L31M/V/F	
V107I	H58D (GT1a only)	
S122A/G/R	Y93C/H/N/S (GT1a only)	
I132V		
R155X		
A156S/T/V/F/G		
V158I		
D168X		
I/V170A/F/T/V		
M175L		

Table S4. A summary of the mean number of sequence reads and wildtype amino acid counts for the two NS5B RAS within the overlapping region obtained by WG and MiDi amplicons: S282 and L320.

		WG Amplicon		MiDi Amplicon	
**NS5B CODON**	Wildtype Amino Acid Residue	Number of passed reads	Number of reads with wildtype residue (%)	Number of passed reads	Number of reads with wildtype residue (%)
**282**	S	3588	3559 (99.1)	951	945 (99.4)
**320**	L	3899	3876 (99.3)	1401	1394 (99.5)

Genotype-Independent Method (columns) × GT1-Optimized Method (rows)

NS3

	A	C	G	T	R	Y	W	M	K	S	B	D	H	V	N
A	32,075	0	0	0	16	0	6	2	0	0	0	0	1	0	0
C	0	48,835	0	0	0	47	0	3	0	1	0	0	0	0	0
G	0	0	42,991	0	28	0	0	0	0	2	0	0	0	0	0
T	0	0	0	31,319	0	38	1	0	2	0	0	0	0	0	0
R	10	0	14	0	515	0	0	0	0	0	0	0	0	0	0
Y	0	32	0	26	0	1,023	0	0	0	0	0	0	0	0	0
W	4	0	0	1	0	0	41	0	0	0	0	0	0	0	0
M	0	1	0	0	0	0	0	30	0	0	0	0	1	0	0
K	0	0	1	1	0	0	0	0	22	0	0	0	0	0	0
S	0	0	1	0	0	0	0	0	0	24	0	0	0	0	0
B	0	0	0	0	0	0	0	0	0	0	1	0	0	0	0
D	0	0	0	0	0	0	0	0	0	0	0	1	0	0	0
H	0	0	0	0	0	0	0	0	0	0	0	0	2	0	0
V	0	0	0	0	0	0	0	0	0	0	0	0	0	1	0
N	0	0	0	0	0	0	0	0	0	0	0	0	0	0	0

NS5A

	A	C	G	T	R	Y	W	M	K	S	B	D	H	V	N
A	22,411	0	0	0	21	0	1	0	0	0	0	0	0	0	0
C	0	33,734	0	0	0	20	0	2	0	1	0	0	0	0	0
G	0	0	32,285	0	16	0	0	0	0	2	0	0	0	0	0
T	0	0	0	21,658	0	21	1	0	0	0	0	0	0	0	0
R	18	0	13	0	521	0	0	0	0	0	0	0	0	0	0
Y	0	19	0	23	0	617	0	0	0	0	0	0	0	0	0
W	1	0	0	2	0	0	32	0	0	0	0	0	0	0	0
M	2	1	0	0	0	0	0	52	0	0	0	0	0	0	0
K	0	0	0	0	0	0	0	0	16	0	0	0	0	0	0
S	0	1	0	0	0	0	0	0	0	26	0	0	0	0	0
B	0	0	0	0	0	1	0	0	0	0	1	0	0	0	0
D	0	0	0	0	0	0	0	0	0	0	0	1	0	0	0
H	0	0	0	0	0	0	0	0	0	0	0	0	1	0	0
V	0	0	0	0	0	0	0	0	0	0	0	0	0	1	0
N	0	0	0	0	0	0	0	0	0	0	0	0	0	0	30

NS5B

	A	C	G	T	R	Y	W	M	K	S	B	D	H	V	N
A	20,071	0	0	0	12	0	1	0	0	0	0	0	0	0	0
C	0	23,254	0	0	0	14	0	0	0	0	0	0	0	0	0
G	0	0	22,983	0	13	0	0	0	1	1	0	0	0	0	0
T	0	0	0	16,665	0	13	0	0	0	0	0	0	0	0	0
R	8	0	5	0	264	0	0	0	0	0	0	0	0	0	0
Y	0	10	0	5	0	380	0	0	0	0	0	0	0	0	0
W	0	0	0	1	0	0	9	0	0	0	0	0	0	0	0
M	0	0	0	0	0	0	0	16	0	0	0	0	0	0	0
K	0	0	0	0	0	0	0	0	8	0	0	0	0	0	0
S	0	0	0	0	0	0	0	0	0	11	0	0	0	0	0
B	0	0	0	0	0	0	0	0	0	0	0	0	0	0	0
D	0	0	0	0	0	0	0	0	0	0	0	0	0	0	0
H	0	0	0	0	0	0	0	0	0	0	0	0	2	0	0
V	0	0	0	0	0	0	0	0	0	0	0	0	0	0	0
N	0	0	0	0	0	0	0	0	0	0	0	0	0	0	0

Figure S1. Accuracy nucleotide concordance between GT1-optimized and the genotype-independent sequencing assays. Matrices depicting nucleotide concordance between sequences collected by two different amplification and sequencing methods. Concordant nucleotide calls across methods/replicates are highlighted in green. Partially discordant nucleotides resulting from differences in mixture calling (i.e. one method detected a mixture, while the other detected a component thereof) are highlighted in yellow.

Genotype-Independent Method

Replicate 2

Genotype-Independent Method Replicate 1

NS3

	A	C	G	T	R	Y	W	M	K	S	B	D	H	V	N
A	30,198	0	0	0	24	0	1	0	0	0	0	0	0	0	0
C	0	45,770	0	0	0	71	0	11	0	1	0	0	0	0	0
G	0	0	40,303	0	48	0	0	0	1	15	0	0	0	0	0
T	0	0	0	29,461	0	46	3	0	2	0	0	0	0	0	0
R	57	0	68	0	404	0	0	0	0	0	0	0	0	0	0
Y	0	146	0	98	0	790	0	1	0	0	0	0	0	0	0
W	8	0	0	3	0	0	30	1	0	0	0	0	0	0	0
M	4	6	0	0	0	0	0	25	0	0	0	0	1	0	0
K	0	0	5	3	0	0	0	0	15	0	0	0	0	0	0
S	0	3	5	0	0	0	0	0	0	19	0	0	0	0	0
B	0	0	0	0	0	0	0	0	1	0	0	0	0	0	0
D	0	0	0	0	0	0	0	0	0	0	0	1	0	0	0
H	0	1	0	0	0	0	0	0	0	0	0	0	3	0	0
V	0	0	0	0	0	0	0	0	0	0	0	0	0	1	0
N	0	0	0	0	0	0	0	0	0	0	0	0	0	0	0

NS5A

	A	C	G	T	R	Y	W	M	K	S	B	D	H	V	N
A	20,249	0	0	0	28	0	0	2	0	0	0	0	0	0	0
C	0	30,472	0	1	0	28	0	1	0	2	0	0	0	0	0
G	0	0	29,135	0	28	0	0	0	3	2	0	0	0	0	0
T	0	0	0	19,548	0	34	4	0	2	0	0	0	0	0	0
R	50	0	44	0	414	0	0	0	0	0	0	0	0	0	0
Y	0	62	0	45	0	491	0	0	0	0	1	0	0	0	0
W	0	0	0	5	0	0	24	0	0	0	0	0	0	0	0
M	7	6	0	0	0	0	0	37	0	0	0	0	0	0	0
K	0	0	2	1	0	0	0	0	11	0	0	0	0	0	0
S	0	1	5	0	0	0	0	0	0	21	0	0	0	0	0
B	0	0	0	0	0	0	0	0	0	0	1	0	0	0	0
D	0	0	0	0	0	0	0	0	0	0	0	1	0	0	0
H	0	0	0	0	0	0	0	0	0	0	0	0	1	0	0
V	0	0	0	0	0	0	0	1	0	0	0	0	0	0	0
N	0	0	0	0	0	0	0	0	0	0	0	0	0	0	30

NS5B

	A	C	G	T	R	Y	W	M	K	S	B	D	H	V	N
A	17,663	0	1	0	24	0	0	5	0	0	0	0	0	0	1
C	1	20,425	0	1	0	32	0	0	0	0	0	0	0	0	5
G	2	0	20,200	0	17	0	0	0	1	0	0	0	0	0	4
T	0	0	0	14,633	0	18	2	0	0	0	0	0	0	0	2
R	25	0	22	0	193	0	0	0	0	0	0	0	0	0	1
Y	0	34	0	38	0	270	0	0	0	0	0	0	0	0	0
W	0	0	0	1	0	0	6	0	0	0	0	0	0	0	0
M	1	2	0	0	0	0	0	10	0	0	0	0	0	0	0
K	0	0	2	0	0	0	0	0	5	0	0	0	0	0	0
S	0	0	2	0	0	0	0	0	0	7	0	0	0	0	0
B	0	0	0	0	0	0	0	0	0	0	0	0	0	0	0
D	0	0	0	0	0	0	0	0	0	0	0	0	0	0	0
H	0	0	0	0	0	0	0	0	0	0	0	0	1	0	0
V	0	0	0	0	0	0	0	0	0	0	0	0	0	0	0
N	0	0	0	0	0	0	0	0	0	0	0	0	0	0	0

Figure S2. Nucleotide concordance between accuracy replicates (N=93 samples). Matrices depicting nucleotide concordance between sequences collected by two replicates of the genotype-independent method. Concordant nucleotide calls across methods/replicates are highlighted in green. Partially discordant nucleotides resulting from differences in mixture calling (i.e. one method detected a mixture, while the other detected a component thereof) are highlighted in yellow. Completely discordant basecalls are highlighted in red.

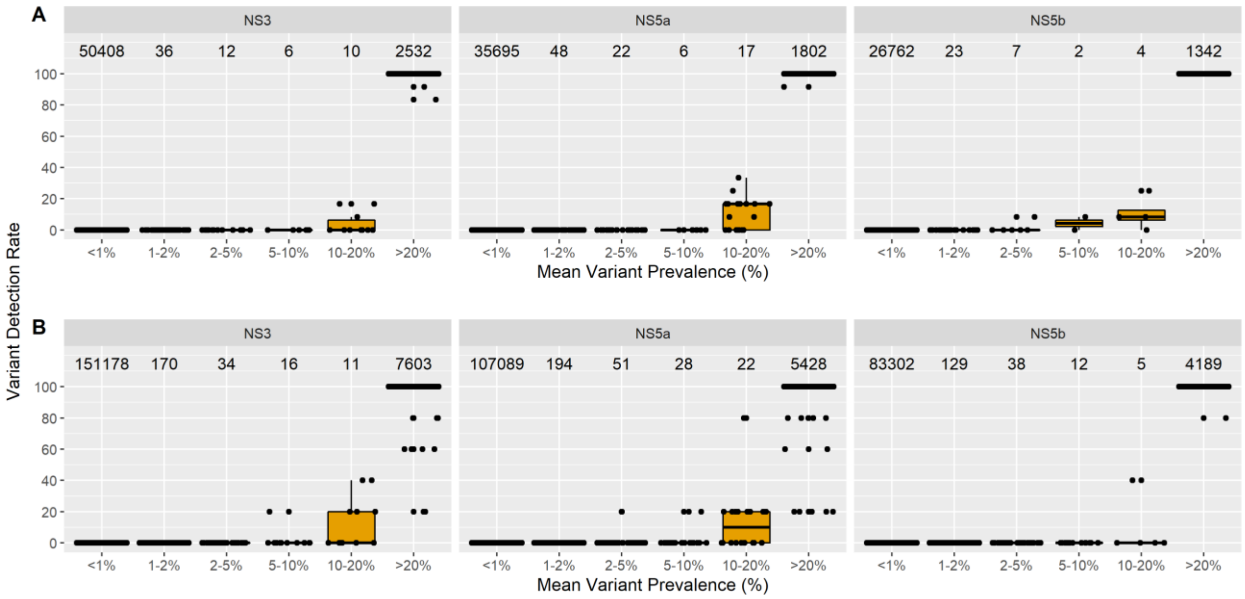

Figure S3. Repeatability and Reproducibility of amino acid substitution detection amino acid substitutions. Substitutions observed at a prevalence ≥20% in a given replicate were considered "detected." Variant detection rate (defined as the % of replicates per sample, in which a substitution was detected) is categorized by expected substitution prevalence (defined as the mean prevalence of a substitution across all replicates of a sample). Replicate testing of each sample began from the same RNA extract; all steps beginning from the RT-PCR were repeated. Numbers indicated in red represent the total number of substitutions in each bin. (A) Repeatability of amino acid substitutions was determined using four samples (3 GT1a, 1 GT1b) in 12 replicates processed on a single separate MiSeq run. (B) Reproducibility of amino acid substitutions was determined using 12 samples (10 GT1a, 1 GT1b, 1 GT3) in 5 replicates processed on separate days on five separate MiSeq runs.
